# Living on the Edge: Demography of the Slender-Billed Gull in the Western Mediterranean

**DOI:** 10.1371/journal.pone.0092674

**Published:** 2014-03-24

**Authors:** Ana Sanz-Aguilar, Giacomo Tavecchia, Isabel Afán, Francisco Ramírez, Aggeliki Doxa, Albert Bertolero, Carlos Gutiérrez-Expósito, Manuela G. Forero, Daniel Oro

**Affiliations:** 1 Department of Conservation Biology, Estación Biológica de Doñana (CSIC), Sevilla, Spain; 2 Population Ecology Group, Institut Mediterrani d’Estudis Avançats, IMEDEA (CSIC-UIB), Esporles, Spain; 3 Laboratorio de SIG y Teledetección (LAST-EBD), Estación Biológica de Doñana (CSIC), Sevilla, Spain; 4 Institut des Sciences de l’Evolution, UMR 5554, CNRS, Université de Montpellier 2, Montpellier, France; 5 Unitat d’Ecosistemes Aquatics, Institut de Reserca i Tecnologie Agroalimentaries, Sant Carles de la Ràpita, Spain; Norwegian Polar Institute, Norway

## Abstract

Small and peripheral populations are typically vulnerable to local extinction processes but important for the metapopulation dynamics of species. The Slender-billed gull (*Chroicocephalus genei*) is a long-lived species breeding in unstable ephemeral coastal habitats. Their Western Mediterranean populations are relatively small and represent the edge of their global geographical distribution. At a local scale, using long-term data (14 years) on annual breeding success and capture-resights of marked individuals, we estimated and compared the vital rates and evaluated the connectivity of two Spanish populations (Ebro Delta and Doñana) varying in their local environmental conditions. At a metapopulation scale, we analyzed 22 years of data on breeding numbers to predict their future prospects by means of population demographic models. Local survival and breeding success of gulls from the Ebro Delta was lower than those from Doñana, which is likely the result of higher permanent emigration and/or winter mortality in the former. Gulls from the Ebro Delta wintered mostly in Mediterranean areas whereas those from Doñana did so in Atlantic coasts, where food availability is higher. Whereas adult local survival was constant, juvenile local survival showed temporal parallel variations between colonies, probably related to natal dispersal to other breeding colonies. Our results suggested that dispersal was higher at the Ebro Delta and gulls emigrating from their natal colonies settled preferentially in close patches. We found large fluctuations in breeding numbers among local populations probably related to the fact that the Slender-billed gull is a species adapted to unstable and unpredictable habitats with high abilities to disperse between suitable patches depending on environmental stochastic conditions during breeding.

## Introduction

Survival, reproduction an dispersal processes drive local population dynamics and metapopulation functioning [1], [2]. Populations at the boundaries of species distributions are often small and show substantial demographic variation over time compared to those occurring at the distribution core [3], [4]. Similarly, species breeding in unstable and ephemeral habitats typically show large fluctuations in population numbers and productivity linked to temporal environmental variation in habitat quality [5], [6]. Adverse environmental conditions may decrease individual survival, reduce breeding output and enhance dispersal probability [3], [7]. Consequently, small and peripheral populations may be more likely to go extinct due to demographic and environmental stochasticity [4], [8] and dispersal processes will become essential for the maintenance of metapopulations [1]. Given the current scenarios of global change, the study of demographic dynamics at multiple populations including marginal or peripheral populations is of special interest for evolutionary and conservation biologists [3], [9]–[12]. In fact, peripheral populations are often genetically different from central populations and may be major contributors to evolutionary changes in the response to environmental changes, such as the global warming or habitat fragmentation [9].

The Slender-billed gull (*Chroicocephalus genei*) is a medium-sized seabird species typically breeding in unstable and ephemeral habitats (e.g. coastal marshes and brackish lagoons inshore) that presents a scattered breeding distribution from Senegal and Mauritania, to Western India [13]. During the second half of the 20th century the species colonized the western part of the Mediterranean [14], breeding in small to medium size colonies (<1000 breeding pairs) in Morocco, Spain, France and Italy [15] and in larger colonies (around 6000 breeding pairs) in Tunisia [16]. Several authors agree that the general growth of main Mediterranean colonies at the western edge of their geographical distribution during the last decades has been likely the result of immigration from the core large Eastern colonies [14], [17]. In fact, a recent study by Doxa et al. (2013) revealed that about 10% of annual breeding individuals at French colonies may be immigrants because the intrinsic local dynamics (low fecundity and survival) could not explain the observed breeding numbers. This was the first study providing estimates of local survival and recruitment probability for the species (i.e., at France) but the extent of spatial variation on demographic parameters (i.e., other local populations), the connectivity between local populations and their overall metapopulation dynamics remains largely unknown [18]. Probability of reproduction and breeding success in dense and synchronous breeding colonies is highly variable and influenced by the availability of feeding resources [17], [19]. As a long lived species, adult Slender-billed gull survival is expected to show low temporal and spatial variability [20]–[22]. However, environmental factors such as climatic conditions or food availability mediated by fishery practices both at breeding and winter quarters may influence local survival generating between population differences, as it has been described for other gull species [23]–[26]. Moreover, dispersal processes in this highly mobile species [27] may also affect the estimation of survival because permanent emigration from a local population and mortality are confounded, leading to a decrease in apparent survival and to biased estimates of true survival [18], [24], [28]. Although dispersal in long-lived birds take place mainly at juvenile stage [29], [30], breeding dispersal also occurs, especially among species breeding in unstable and ephemeral environments [31], [24]. Deterioration of breeding habitat trigger dispersal as a response to a poor breeding output [24], [32]. Settlement decisions are usually related to distance, presence of conspecifics and/or heterospecifics and quality of the patch [31], [24], [33]. Consequently, higher local survival and lower emigration rates would be expected in those colonies exhibiting higher breeding productivity. The Slender-billed gull is a species typically showing complex population dynamics at a local scale [17], [18]. The extent of coupling between local and regional population dynamics in response to common or differential environmental factors and dispersal processes will likely influence the future persistence of the species [2], [34], [35]. Thus, comparative studies of local population variation in demographic parameters and population dynamics are necessary to understand population functioning and design effective conservation plans [12], [11], [36]–[38].

**Figure 1:**
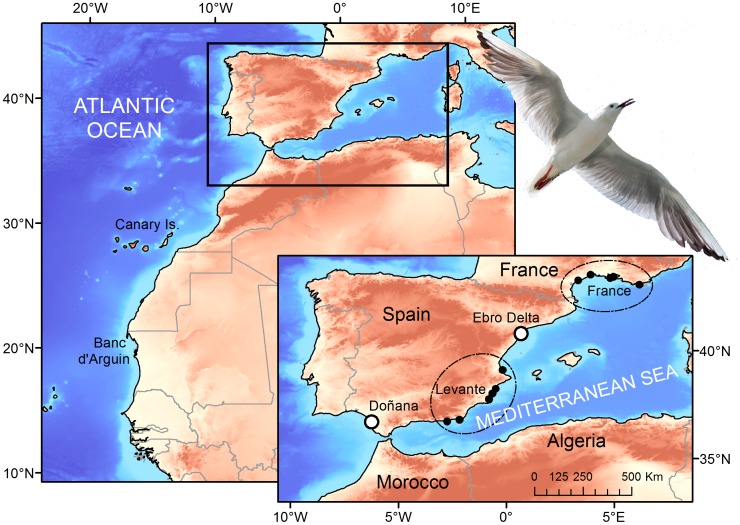
Location of Slender-billed gull Spanish and French breeding colonies.

Here we studied the vital rates, regional connectivity and population dynamics of Slender-billed gulls born at two Spanish colonies 750 km apart (the Ebro Delta and Doñana) located at the edges of their Mediterranean distribution (Fig. 1). The specific objectives of our study were: I) to estimate and compare breeding success and local survival of Slender-billed gulls born at the Ebro Delta and Doñana areas, II) to evaluate the effects of environmental conditions during breeding on local survival, III) to study the extent of wintering and breeding spatial movements of individuals marked at the two studied populations; and IV) to evaluate the breeding population trends, population numbers and future population prospects at both local and regional scale (the Western Mediterranean) over the last two decades.

## Methods

### Ethics Statement

The authors declare that all animals were handled in strict accordance with good animal practice as defined by the current European legislation, and all animal work was approved by the respective regional committees for scientific capture (Consejería de Medio Ambiente de la Junta de Andalucía, Sevilla, Spain and Parc Natural del Delta de l’Ebre,Generalitat de Catalunya). All necessary permits were obtained for the described field studies (provided by “Dirección General de Gestión del Medio Natural- Junta de Andalucía” and “Dirección General de Espacios Naturales y Participación Ciudadana- Junta de Andalucía” and “Direcció General del Medi Natural-Generalitat de Catalunya”).

### The Breeding Colonies in the Western Mediterranean

In the Western Mediterranean, the Slender-billed gull breeding colonies are located on dikes and islets inside saltpans, coastal lagoons and marshes. The precise spatial location of Slender-billed gull breeding colonies typically varies over time and small scale movements of the species are frequent [17]–[19]. Gulls breed in particularly dense colonies mostly in association with other gull and wader species; lay mostly 2–3 eggs and chicks are grouped in crèches soon after fledgling. Regular reproduction of species in the Western Mediterranean began in the 70′s [15]. In Spain, the first and largest colonies were established in the Ebro Delta in north eastern Spain and the Natural Area of Doñana (hereafter Doñana) on the South-Western Atlantic coast (Fig.1). Both the Ebro Delta and Doñana breeding areas included up to three different sites where the species has bred during the period considered in this study [17], [19]. However, the species has also been breeding over the last 20 years in the Levante Region on the eastern Spain coast including six different breeding areas (Fig. 1). In Southern France the species breeds regularly in the Rhône Delta and sporadically in other five areas including saltpans and wetlands [18].

### Vital Rates at Ebro Delta and Doñana Colonies

The total number of nests and fledglings in the crèche at the end of the chick-rearing period was counted from 1995 to 2008 at Ebro Delta and Doñana (see details in [17], [19]). Annual breeding success was estimated as the ratio between number of fledglings (counted during ringing operation of the crèche) and number of nests counted (assuming no sampling errors in counts). We assume that fledgling mortality after fledgling counts is negligible, as reported by Chokri et al. (2011) [39].

Local survival of individuals fledged at both colonies was estimated by means of individual monitoring and capture-recapture models [28]. From 1995 to 2008 at Doñana and from 1998 to 2005 at Ebro Delta 70% and 52% of the fledglings were captured and marked individually with plastic rings engraved with three digit code. Ring codes can be read from distance using telescopes. We considered 5042 and 2455 observations of 3303 and 1662 individuals at Doñana and Ebro Delta, respectively. At both sites, observations of marked individuals were taken from April to July at breeding areas until 2008. Capture-recapture models were built separately for each data-base using program E-SURGE 1.4.4. [40]. The few observations from individuals resighted at the other colony (n = 25, Table 1) were not taken into account. The small number prevented to obtain a robust estimate of between colonies movements using capture-mark-recapture models. We considered as a starting point a general model including the effects of time (i.e., year) and age (3 and 4 age classes, respectively) in local survival and resighting probabilities. The choice of 3 age classes for modeling survival was based on previous results for the species [18]. However, we considered 4 age classes for modeling resighting probabilities in the initial model because in long lived species, accession to reproduction that influences resighting probabilities may be delayed [37]. We assessed the goodness-of-fit of the general Cormarck Jolly Seber model, ‘CJS’, [28] for each cohort-by-colony using program U-CARE 2.2.2. [41]. Model selection was based on the Akaike’s Information Criterion adjusted for small sample size,AICc; [42]. For both datasets we initially tested the effects of time and simpler and more complex age structures on resighting probabilities. Once the structure of resighting probabilities that minimized AICc was selected we tested the effect of time and age structure on survival. Finally, using the structure of the model with the lowest AICc we tested the effects of variation of marshland inundation at Doñana and fish landings at Ebro Delta, as proxies to locally available food resources (see below), on gull local survival. The effect of these covariates was assessed using the analysis of deviance, ANODEV, [43]. This method allows testing if the variability of the parameter considered explained by the covariate is statistically significantly different from that explained by the time dependent model [44]. We additionally calculate the percentage of variation that was explained by a covariate (r^2^) [44].

**Table 1:** Modelling resighting and local survival probabilities of Slender-billed gulls at the Ebro Delta.

*Modeling resighting probabilities at Ebro Delta (survival was modeled as* a(1,2, ≥3).t)							
*Model*	*Resighting*	*np*	*Dev*	*AICc*	*ΔAICc*		
1EB	a(1,2,≥3).t	45	4014.01	4105.75	9.02		
**2EB**	**a(1,2,3,** ≥**4).t**	**52**	**3990.41**	**4096.72**	**0**		
4EB	t	31	4089.16	4151.98	55.26		
5EB	a(1,2,3,4, ≥5).t	58	3988.82	4107.69	10.97		
6EB	a(1,2,3, ≥4)+t	35	4031.32	4102.37	5.64		
7EB	a(1,2,3, ≥4)	27	4171.76	4226.39	129.67		
***Modeling survival probabilities at Ebro Delta (resighting was modeled as a(1,2,3, ≥4).t)***							
***Model***	***Survival***	***np***	***Dev***	***AICc***	***ΔAICc***	***ANODEV***	***r^2^***
2EB	a(1,2, ≥3).t	52	3990.41	4096.72	7.17		
8EB	a(1,2,3, ≥4).t	57	3981.91	4098.69	9.14		
9EB	a(1, ≥2).t	46	3998.76	4092.57	3.01		
10EB	t	40	4077.29	4158.66	69.11		
11EB	a(1, ≥2)+t	41	4007.04	4090.47	0.92		
12EB	a(1, ≥2)	33	4026.82	4093.75	4.20		
**13EB**	**a(1).t/a(**≥**2)**	**40**	**4008.18**	**4089.55**	**0**		
14EB	a(1)/a(≥2).t	40	4012.89	4094.26	4.71		
15EB	a(1).FISH/a(≥2)	34	4024.16	4093.15	3.60	F_(1,6)_ = 1.00 n.s.	0.14
16EB	a(1).t/a(≥2).FISH	41	4008.04	4091.48	1.92	F_(1,5)_ = 0.08 n.s.	0.02
17EB	a(1, ≥2).FISH	34	4026.06	4095.05	5.50	F_(1,6)_ = 0.28 n.s.	0.04

Notation, np: number of estimable parameters; Dev: relative deviance; AICc: Akaike information criterion corrected for small sample size; ΔAICc: the AICc difference between the current model and the one with the lowest AICc value; ‘t’ indicates a time effect; a() indicate the number of age classes considered; ‘FISH’ is the fish landings covariate; ‘/’ separates different effects considered independent for each age class; ‘+’ denotes an additive effect and ‘.’ interaction. Results of the ANODEV test are presented, n.s. indicate not significant effect. r^2^ indicates the proportion of temporal variation of survival explained by the covariate. The model with the lowest AICc is in bold.

Finally, using the retained structure of resighting probabilities and the age structure retained for survival in the previous analyses, we built some additional joined models testing for differences in local survival between colonies (colony effect models vs. constant models) and the effect of winter North Atlantic Oscillation index, NAO [45], (as a proxy of large scale environmental variation on both juvenile and/or adult survival. The NAO index measures differences in sea level and atmospheric pressure between the Azores and Iceland [45].

### Local Environmental Covariates at Ebro Delta and Doñana Breeding Areas

At the Ebro Delta Slender-billed gulls forage at saltpans and coastal marine areas [46]. We collected data on annual fish landings at Sant Carles de la Ràpita harbour (South Ebro Delta) during the month of April as a measure of fish resources availability at the Ebro Delta breeding area during the breeding season (see details in [47]). Following Ramírez et al. (2012), we calculated average inundation levels at the marshland of Doñana for the 1998 to 2008 breeding periods (April to July). Natural marshes constitute the main foraging habitat for avian species inhabiting and breeding at Doñana [19], [48]–[50]. Moreover, and provided that alternative, man-made habitats (i.e., saltworks and fishfarm) showed relatively constant hydrological regimens [19], inter-annual fluctuations in marshland extent could be also considered as a reliable proxy to inter-annual changes in overall availability of local food resources [19], [50]. Raw data on inundation levels were extracted from Landsat, TM and ETM+ images (see details in [51]), whereas missing data were filled with piecewise cubic Hermite interpolations [19].

### Large Scale Environmental Covariates

We used the global indices of winter NAO available at http://www.cgd.ucar.edu/cas/jhurrell/indices.html as a covariate to investigate the possible association between survival and environmental conditions (i.e., rainfall levels). This index has been showed to influence juvenile wintering migration in the Greater Flamingo *Phoenicopterus ruber* [52], a species sharing breeding habits and wintering sites with the Slender-billed gull. The winter NAO drives the precipitation variability during the wettest months in the Mediterranean and has a direct impact on droughts [53]. During its negative phase, NAO steers the storm-track towards southern Europe, thus increasing precipitation observed over the Mediterranean, and vice versa [50].

### Spatial Movements of Marked Individuals

Data on spatial distribution of individuals marked as fledglings at Ebro Delta and Doñana during wintering (from November to February, N = 570 resightings) and breeding seasons (from April to July, N = 9112 resightings) was obtained by the resightings of 280 (wintering) and 2064 (breeding) marked individuals reported by research groups and amateur ornithologists.

In addition, we used the approximation of Balkiz et al. (2007) to obtain an estimation of the number (and proportion) of breeders in a given colony, *s,* originated from the site, *r,* in a given year. This approach is based on correcting the observed number of immigrants at *s* each year by the proportion of birds marked in *r* and the annual recapture probability at *s* [54].

### Population Dynamics

To evaluate breeding population trends of the species at a regional scale (i.e., the Western Mediterranean) we collected additional information of breeding numbers in Southern France and Levante Region (Fig. 1). Data available was the total number of nests counted in Spanish and French breeding colonies from 1991 to 2012 (except for 1996 in France, see details in [14], [17]–[19], [55], [56]). Annual population growth rates (λ_t_) of Slender billed gulls breeding populations at each breeding area (Fig. 1) and in the whole Western Mediterranean was calculated as λ_t_ = N_t+1_/N_t_, using censuses of nests (N). In addition, we calculated the stochastic population growth (λ_s_) and its confidence interval by the mean of a linear regression procedure (see details in [2], [57], Supporting Information S2) to determine the future chances of population growth or decline [2]. This method allows λ_s_ estimation when censuses were not taken at equal time intervals (i.e., when there are gaps in time series) by regressing the log population growth rate λ_s_ over a time interval against the amount of time elapsed [57]. Values of λ higher or equal than 1 predict that most future population trajectories will grow or persist in their current state. However, if the lower limit of the confidence interval for λ_s_ is below 1 the population would tend to decline over the long term [2]. Note that for Levante Region, λ_s_ was calculated for the period in which gulls regularly bred in the region (1994 to 2012).

In addition, we use the deterministic matrix population model proposed by Doxa et al. (2013) to estimate the asymptotic population growth rate at Donaña and Ebro colonies during the period 1998–2004 and 1995–2004, respectively using the respective annual estimates of fecundity (i.e., number of chicks fledged per breeding pair) and of local juvenile and adult survival probabilities (see above). We assumed the same recruitment probability as estimated for the French population [18]. The estimates of population growth rates obtained by the matrix population model were compared with those based on nest counts (see above).

## Results

### Vital Rates at Ebro Delta and Doñana Colonies

**Figure 2:**
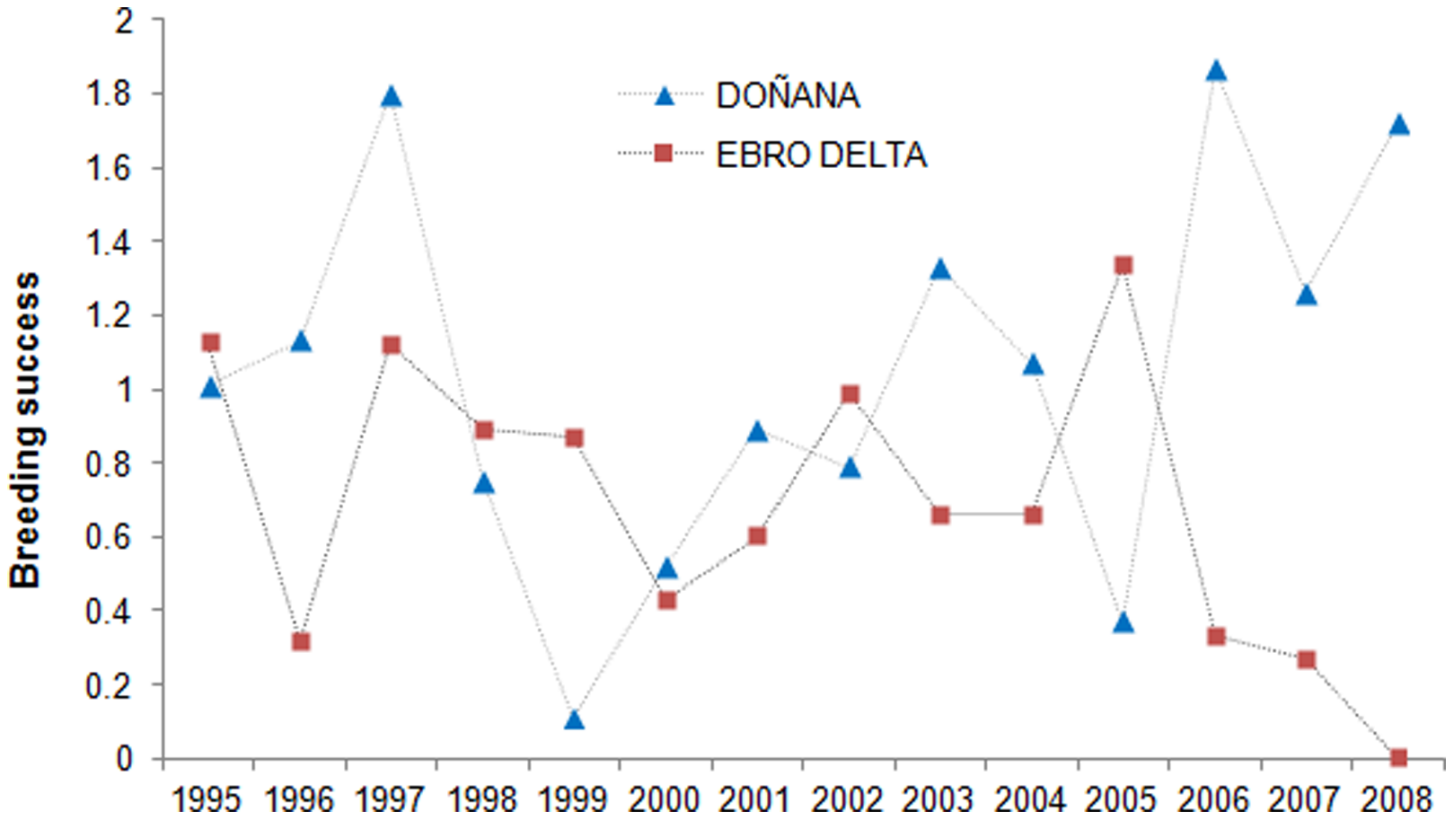
Annual estimates of Slender-billed gull breeding success. Breeding success was estimated as the ratio between the number fledglings and the number of nests counted at Ebro Delta and Doñana colonies from 1995 to 2008.

Annual breeding success of Slender-billed gulls was highly variable at both breeding areas ranging from complete breeding failure to 1.9 fledglings per nest (Fig. 2). Mean breeding success was lower at the Ebro Delta than at Doñana: 0.69 (SE = 0.10) and 1.04 (SE = 0.14), respectively (t = 2.04, df = 26, p = 0.05).

**Figure 3:**
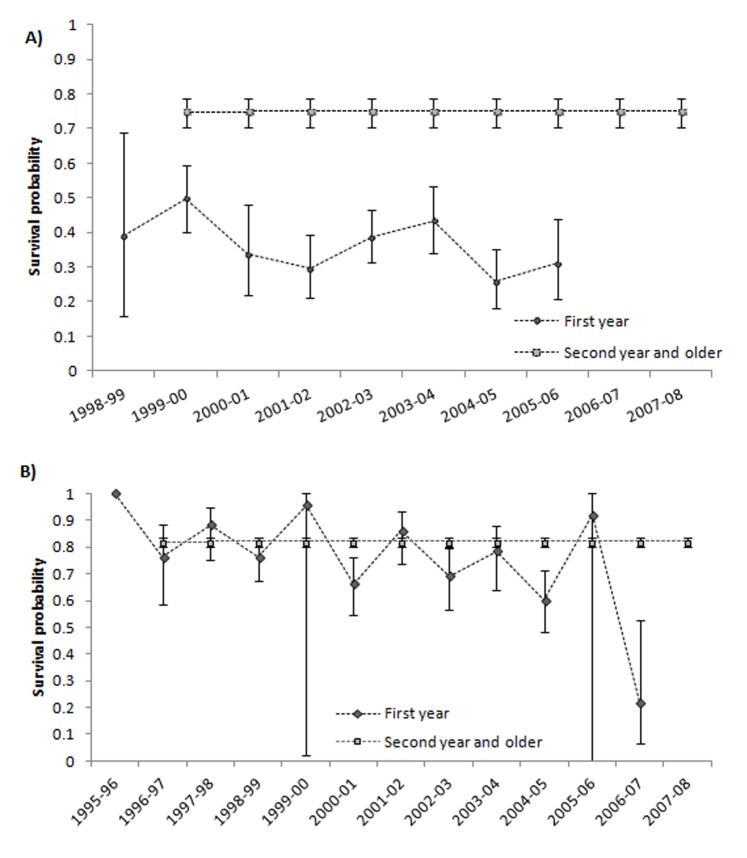
Age-dependent survival probabilities (and 95% CI) of Slender billed gulls. Estimates of Slender billed gulls survival at Ebro Delta (A) and Doñana (B) during the study period from models 13EB and 13DO (Tables 1–2).

Goodness of fit tests of the CJS model for each cohort and colony were not statistically significant indicating a good fit of the general models (Global test for Ebro Delta χ^2^ = 41.70, d.f. = 62, P = 0.978 and Doñana χ^2^ = 114.50, d.f. = 161, P = 0.998). At the Ebro Delta (‘EB’ in model notation), the best supported model (Model 2EB, Table 1) indicated that resighting probabilities varied over time and age (i.e., 4 age classes: first year, second year, third year and older gulls) (Fig. S1 in Supporting Information S1). At Doñana (‘DO’ in model notation), the model with the lowest AICc showed that resighting probabilities varied over time and between first year and older gulls (Model 3DO, Table 2, Fig. S1 in Supporting Information S1). Resighting probabilities generally increased with individual’s age at both colonies (Fig. S1 in Supporting Information S1). As no other models considering different structures of resighting were tied in terms of AICc, we considered the structure of model 2EB at Ebro Delta (Table 1) and model 3DO at Doñana (Table 2) to modeling survival probabilities at both colonies. Results of model selection showed a similar structure of survival probabilities in both colonies (Models 13EB and 13DO, Tables 1–2): temporal variation for first year juvenile gulls and constant values for older birds (Fig. 3). However, mean estimates of survival were different between colonies for both age classes (Adults, Model 1B vs. 2B and Juveniles, Model 3B vs. 4B, Table 3). Mean local survival at Ebro Delta (1st year 0.38 CI: 0.34–0.42, 2nd year and older 0.76 CI: 0.71–0.80) was lower than at Doñana (1st year 0.78 CI: 0.73–0.82, 2nd year and older 0.83 CI: 0.81–0.85) (Models 12EB and 12 DO, Tables 1–2).

**Table 2:** Modelling resighting and local survival probabilities of Slender-billed gulls at the Doñana breeding area.

*Modeling resighting probabilities at Doñana (survival was modeled as* a(1,2, ≥3).t)							
*Model*	*Resighting*	*np*	*Dev*	*AICc*	*ΔAICc*		
1DO	a(1,2, ≥3).t	69	11009.22	11149.03	84.94		
2DO	a(1,2,3, ≥4).t	77	10916.84	11073.10	9.01		
**3DO**	**a(1,** ≥**2).t**	**58**	**10946.80**	**11064.09**	**0**		
4DO	t	86	10899.41	11074.22	10.14		
5DO	a(1,2,3,4, ≥5).t	48	11219.91	11316.79	252.7		
6DO	a(1,2,3, ≥4)+t	49	11007.13	11106.04	41.96		
7DO	a(1,2,3, ≥4)	38	11258.31	11334.86	270.78		
***Modeling survival probabilities at Doñana (resighting was modeled as a(1, ≥2).t)***							
***Model***	***Survival***	***np***	***Dev***	***AICc***	***ΔAICc***	***ANODEV***	***r^2^***
3DO	a(1,2, ≥3).t	58	10946.80	11064.09	17.96		
8DO	a(1,2,3, ≥4).t	68	10937.27	11075.03	28.91		
9DO	a(1, ≥2).t	48	10953.66	11050.53	4.41		
10DO	t	37	10987.26	11061.79	15.67		
11DO	a(1, ≥2)+t	38	10979.86	11056.41	10.29		
12DO	a(1, ≥2)	27	11008.13	11062.41	16.29		
**13DO**	**a(1).t/a(**≥**2)**	**38**	**10969.57**	**11046.12**	**0**		
14DO	a(≥2).t/a(1)	37	10974.49	11049.01	2.89		
15DO	a(1).MAR/a(≥2)	28	1108.12	1164.42	18.30	F_(1,10)_ = 0.00 n.s.	0.00
16DO	a(1).t/a(≥2).MAR	39	10969.23	11047.81	1.69	F_(1,9)_ = 0.20 n.s.	0.02
17DO	a(1, ≥2).MAR	28	11008.08	11064.38	18.26	F_(1,10)_ = 0.02 n.s.	0.00

Notation, np: number of estimable parameters; Dev: relative deviance; AICc: Akaike information criterion corrected for small sample size; ΔAICc: the AICc difference between the current model and the one with the lowest AICc value; ‘t’ indicates a time effect; a() indicate the number of age classes considered; ‘MAR’ is the marshland inundation covariate; ‘/’ separates different effects considered independent for each age class; ‘+’ denotes an additive effect and ‘.’ interaction. Results of the ANODEV test are presented, n.s. indicate not significant effect. r^2^ indicates the proportion of temporal variation of survival explained by the covariate. The model with the lowest AICc is in bold.

**Table 3:** Modelling local survival probabilities of Slender-billed gulls at the Ebro Delta and Doñana breeding areas.

*Model*	*Predictors of survival*	*np*	*Dev*	*AICc*	*ΔAICc*	*ANODEV*	*r^2^*
1B	a(1).col.t/a(≥2).col	78	14977.75	15135.34	0		
2B	a(1).col.t/a(≥2)	77	14987.04	15142.59	7.28		
3B	a(1, ≥2).col	60	15034.95	15155.89	20.55		
4B	a(1)/a(≥2).col	59	15158.92	15277.83	142.49		
5B	a(1, ≥2).col.t	94	14952.42	15142.72	7.38		
6B	a(1).col.NAO/a(≥2).col	62	15031.40	15156.41	21.07	F_(2,16)_ = 0.53 n.s.	0.06
7B	a(1).col.t/a(≥2).col.NAO	80	14974.05	15135.72	0.38	F_(2,14)_ = 1.20 n.s.	0.15
8B	a(1, ≥2).col. NAO	64	15030.33	15159.40	24.05	F_(4,30)_ = 0.44 n.s.	0.06

Resighting probabilities were modeled following the structure of models 13EB and 13DO for each colony (Tables 2 and 3). Notation, np: number of estimable parameters; Dev: relative deviance; AICc: Akaike information criterion corrected for small sample size; ΔAICc: the AICc difference between the current model and the one with the lowest AICc value; ‘col’ indicates a colony effect; ‘NAO’ is the winter NAO covariate; ‘t’ indicates a time effect; a() indicate the number of age classes considered; ‘/’ separates different effects considered independent for each age class; ‘+’ denotes an additive effect and ‘.’ interaction. The model with the lowest AICc is in bold.

The effect of local (marshland surface at Doñana and fish landings at Ebro Delta) and large scale (winter NAO) covariates on individual local survival was not statistically significant and explained low proportions of the temporal survival variability (Models 15–17, Tables 1–2 and Models 6B–8B, Table 3).

### Spatial Distribution of Marked Individuals during Winter and Breeding Seasons

**Figure 4:**
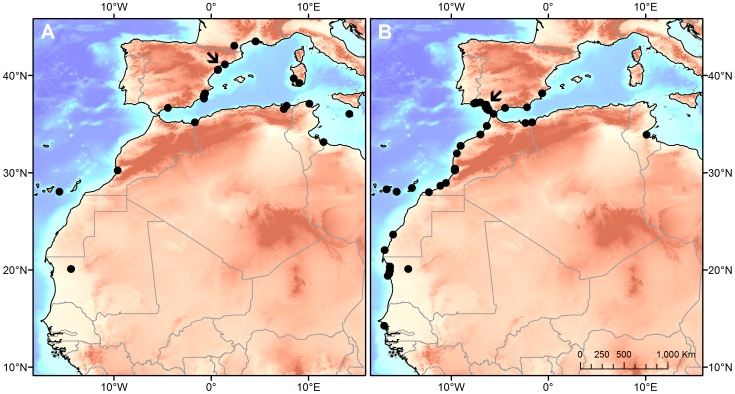
Winter distribution of Slender billed gulls born at Ebro Delta (A) and Doñana (B). Spatial distribution of wintering (from November to February) resightings of Slender billed gulls marked as chicks at Ebro Delta (A) and Doñana (B) colonies (black dots). Arrows indicate the location of the breeding colony of origin.

Gulls marked at Ebro Delta and Doñana wintered in different locations: being gulls from Ebro Delta observed more often within the Mediterranean basin (96.4% observations, N = 56) and gulls from Doñana along the shores of the Atlantic Ocean (91.4% observations, N = 224), mainly at the Gulf of Cadiz (Spain), the Moroccan coast and Banc d’Arguin (Mauritania) (Fig. 4). Individuals from Doñana wintered more southerly than individuals fledged at the Ebro Delta (Fig. 4).

Individuals marked at Ebro Delta and Doñana were resighted during subsequent breeding periods in higher proportions at their natal colonies than elsewhere (79.9% and 98.5% of resightings, respectively; Table S1 in Supporting Information S1). However, individuals from the Ebro Delta were resighted in higher proportions out of their natal areas than individuals from Doñana (41.5% and 4.3% of resightings, respectively; Table S1 in Supporting Information S1), even during the same breeding season (own data). A higher proportion of individuals from the Ebro Delta than from Doñana were resighted in at least two breeding areas during the study period (20.1% and 2.7% resightings, respectively; Table S1 in Supporting Information S1). Observations of movements between Ebro Delta and Doñana were less frequent than observation of movements between the former area and Levante region (Table S1 in Supporting Information S1). Individuals from Doñana were also observed at lower proportions at France than those born at Ebro Delta (Table S1 in Supporting Information S1).

The estimated annual number and proportion of immigrant birds from the alternative colony ranged between 0–3% (Table S2 in Supporting Information S1).

### Western Mediterranean Breeding Population Dynamics

**Figure 5:**
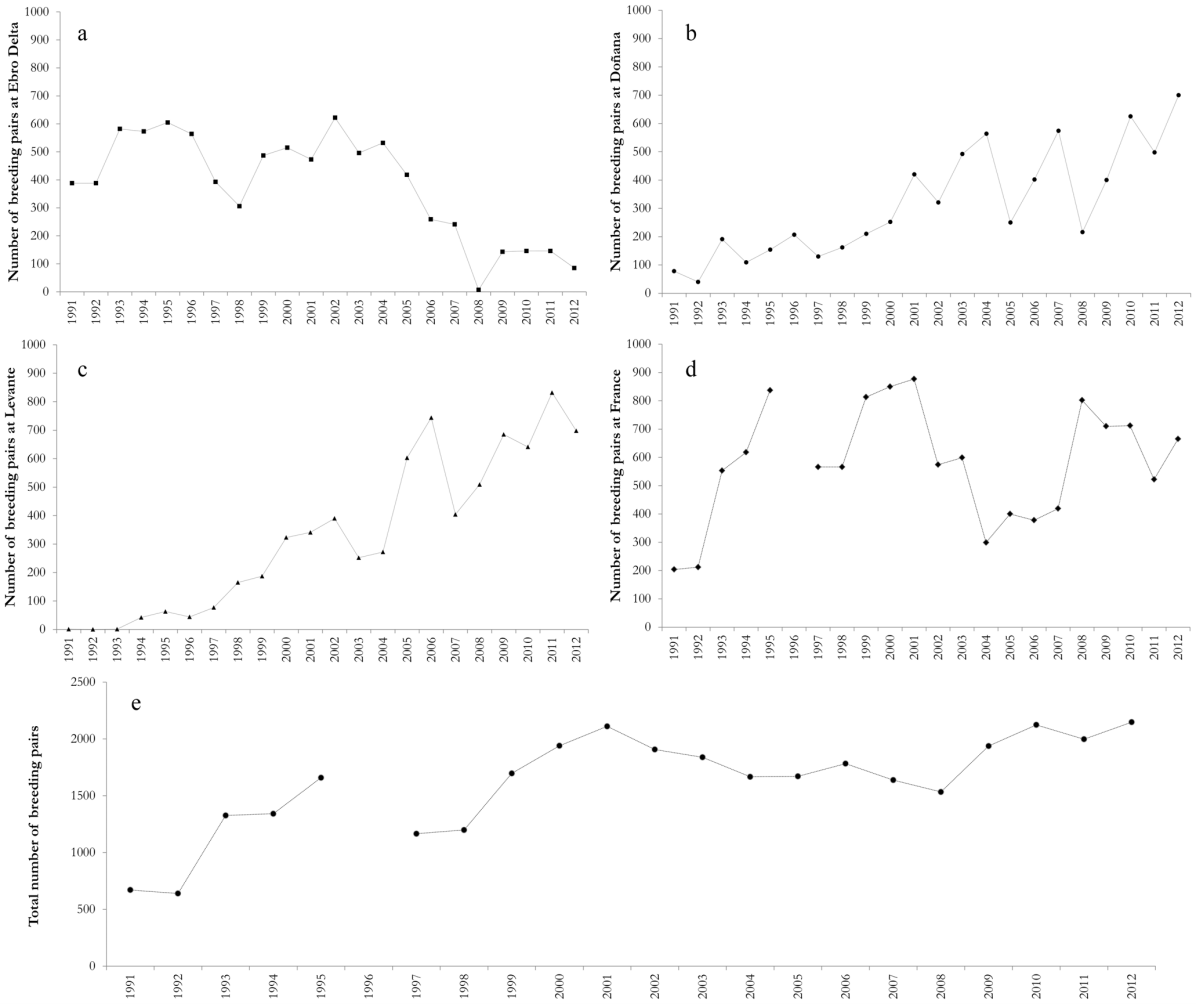
Annual breeding numbers of Slender-billed gulls at Ebro Delta (a), Doñana (b), Levante (c), France (d) and the Western Mediterranean (e). Breeding numbers was defined as the number of nests counted. The Western Mediterranean includes Spanish and French colonies (Fig. 1).

The observed number of Slender billed gulls breeding pairs from 1991 to 2012 in the breeding regions considered showed a high temporal variability with substantial yearly variations (Fig. 5; Table S3 in Supporting Information S1). The Ebro Delta (EB), the largest breeding population in 1991, showed a negative trend and the largest fluctuations of breeding pairs resulting in a λ_EB_ = 0.93 (CI: 0.59–1.47). Populations in Doñana (DO) and Levante (LE) and France (FR) showed positive growth trend during the study period: λ_DO_ = 1.11 (CI: 0.87–1.43), λ_LE_ = 1.17 (CI: 0.98–1.40) and λ_FR_ = 1.06 (CI: 0.91–1.24), respectively. At a broader spatial scale, annual breeding numbers in whole Western Mediterranean region (WM) fluctuated to a lesser extent than in each of the considered breeding populations (Table S3 in Supporting Information S1). The mean stochastic population grow rate was λ_WM_ = 1.06 (CI: 0.97–1.16). In all regions considered the confidence intervals of λ_s_ included the stable value (1) indicating that either growth or decline of populations is possible. However local population extinction in the short term is quite improbable except for the Ebro Delta breeding local population.

**Figure 6:**
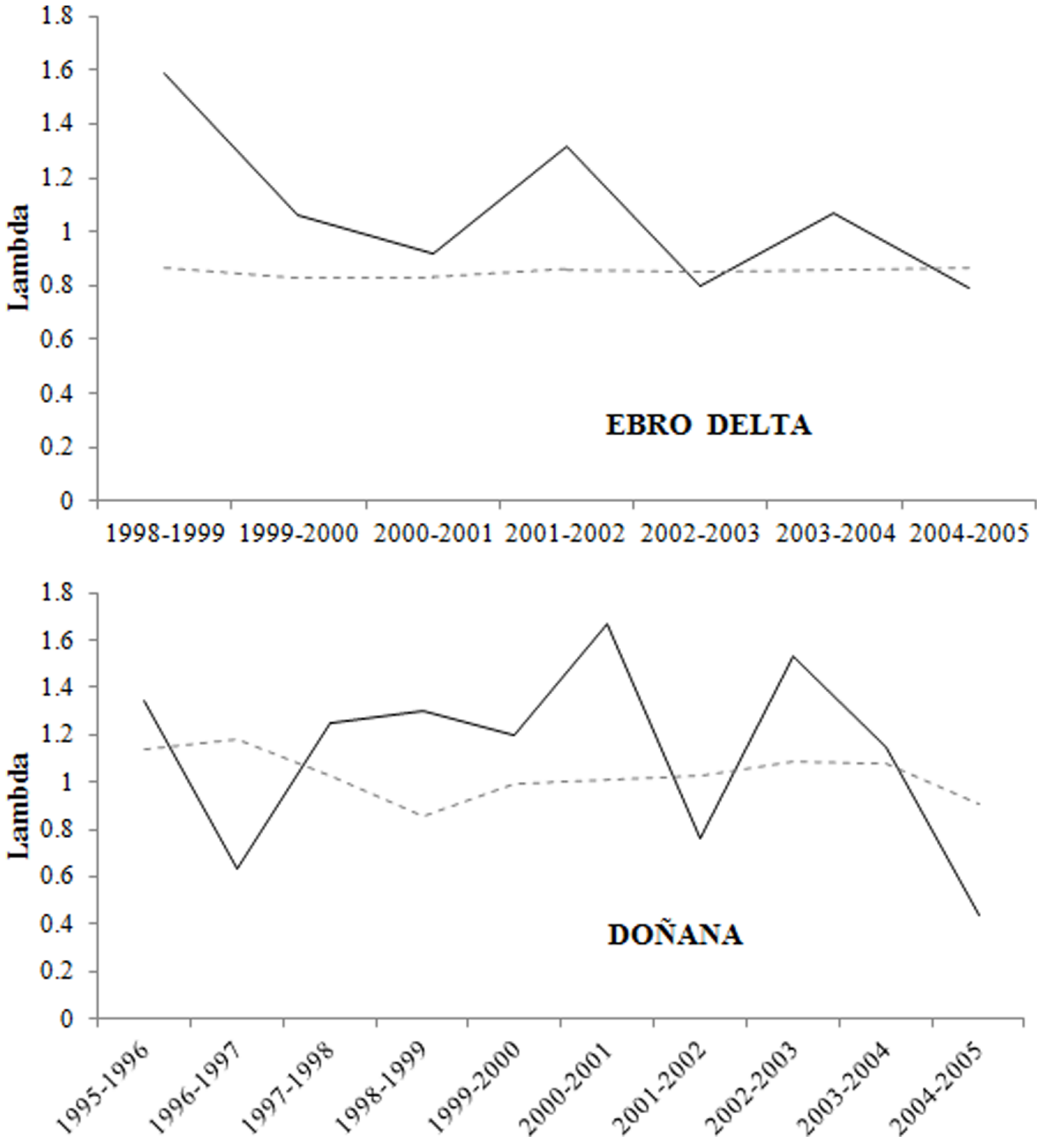
Population growth rates of Slender-billed gulls at Ebro Delta and Doñana colonies estimated by nest counts (solid lines) and by the population model including local demographic parameters (dashed line).

Matrix population models indicated a different long-term dynamics for the two colonies. In Doñana the estimated average population growth rate (geometric mean) during the period 1995–2004 was 1.04, indicating an increase of 4% in the number of breeding pairs, whereas at the Ebro Delta it was 0.85 for the period 1998–2004 suggesting a declining population. However, the observed average population growth rates for the same periods (based on nest counts) were 1.11 and 1.10 for Doñana and the Ebro Delta colony, respectively. The difference between the observed and predicted value of the population growth rate was higher for the Ebro Delta colony (Fig.6).

## Discussion

Studying the extent of variation in key demographic parameters (i.e. reproduction, survival, emigration and immigration) at multiple local populations is essential to understand population dynamics and metapopulation functioning [31], [12], [3], [11]. Unfortunately at the moment, estimates of vital rates from populations at the core range of the Slender-billed gull distribution are not available. Nevertheless, as expected for peripheral populations we found substantial demographic variation (in breeding success and juvenile survival) at both temporal and spatial scales [3], [4]. Overall, mean breeding success of Slender-billed gulls at Doñana (1.04) was higher than at the Ebro Delta (0.69) and higher than at French (0.66); [18] and Tunisian colonies (0.45 early breeders and 0.85 late breeders); [58]. Productivity in this species increases with colony size [59] and food availability [17], [19], but it is also influenced by predators and human disturbance [17]. Doñana and the Ebro Delta are legally protected coastal areas where man-made habitats provide gulls with alternative feeding resource [17], [19], thus likely increasing the species breeding productivity. Further, gull breeding success showed substantial temporal fluctuations at the two studied colonies (Fig. 2) probably due to variations in breeding numbers and local environmental conditions [17], [19]. Both breeding numbers and breeding success of Slender-billed gulls at Doñana were higher than at the Ebro Delta during the last years of study (Fig 2, 5), likely as a result of increasing interferences by competitors (e.g., Audouin’s gulls *Larus audouini* and Yellow legged gulls *Larus michahellis*) which populations has dramatically raised over the last years at the Ebro Delta [60], [61].

Adult survival did not show temporal variations during the study period, as expected for a long-lived species [62]. Local adult survival estimated at Doñana (0.83, CI: 0.81–0.85) was similar to the one at French colonies (0.81, CI: 0.79–0.83, [18]) but higher than the one found at Ebro Delta(0.76, CI: 0.71–0.80). Evidence gathered on individual wintering distribution, indicate that individuals from the two Spanish populations showed different wintering migration quarters: whereas gulls from the Ebro Delta mainly remained at the Mediterranean, those from Doñana moved South to highly productive Atlantic areas [63], [64]. Environmental conditions experienced by individuals from the two populations during the non-breeding season were probably different. Dispersal occurs mainly at juvenile stages [29], [30]. In fact, we only found temporal variation for juvenile local survival and not for adult survival. Resightings out of their natal areas during the breeding season of individuals ringed at the Ebro Delta and Doñana suggest that gulls may be dispersing to French and Levante breeding areas. Accordingly, colonies at Levante Region grew substantially during the study period (Fig. 5c) and population models for the French population suggest that immigration processes in this population play an important role [18]. Data on resightings also suggested that dispersal of gulls from the Ebro Delta was higher than that from Doñana. This result is in accordance with the lower local survival probabilities estimated for juvenile (i.e., first year) gulls from the Ebro Delta. Gulls could be dispersing as a consequence of the lower breeding success experienced at the Ebro Delta and/or being attracted to other neighboring colonies [31], [24], [32], [33], [65]. In contrast, at Doñana, the higher expectances of successful reproduction and the larger distances to other colonies (Fig. 1) could be limiting dispersal processes [31]. Moreover, the higher food availability and the potentially more favorable environmental conditions experienced by individuals from Doñana at their wintering quarters may also explain their higher juvenile and adult local survival [52]. Although several authors agree that Western Mediterranean colonies of Slender-billed gulls were formed by immigration from large colonies at the Black Sea [14], [17], the observed differences in wintering areas between individuals of the Ebro Delta and Doñana suggest an African origin (e.g., Senegal and Mauritania) of gulls emigrating to Doñana [66], [67]. However, further studies (e.g. on genetic diversity) will be necessary to confirm this hypothesis.

Marginal or peripheral populations and species breeding in unstable coastal habitats typically show a dynamic dominated by dispersal processes, massive breeding failures or intermittent reproduction [3], [4], [68], [69]. Accordingly, we found substantial fluctuations in breeding numbers. Although estimated local adult survival in the two studied populations was constant during the study period, temporal variations in breeding success and juvenile local survival (that includes permanent emigration) could be responsible of population fluctuations via a substantial temporal variation in recruitment [70]. In addition, variations in breeding population numbers could also be explained by intermittent breeding due to environmental factors or local disturbances [17], [71]. Whereas the biggest colony at the beginning of the study (Ebro Delta) suffered a drastic reduction in breeding numbers, Doñana and French colonies grew and new colonies have been formed at Levante Region. Intense competition with Audouin’s gulls or Yellow-legged gulls at the Ebro Delta could be causing Slender-billed gulls to disperse to other colonies [18], [61]. In addition, settlement decisions in seabirds are usually related to colony size, distance and breeding performance expectances [31], [33], [65]. Accordingly, we found that gulls were more resighted at nearby colonies than at more distant colonies from their natal site. In fact, birds from the Ebro Delta, where mean breeding success and distance to neighbouring colonies was lower than in Doñana (Fig. 1, 2), were resighthed in other colonies in higher proportions than birds from Doñana. Juvenile survival of individuals of Doñana population, where dispersal may be low, were quite high and similar to adult survival suggesting that the differences between juvenile and adult survival found in the Ebro Delta or France [18] may be partially due to permanent natal dispersal. Population growth rates estimated by nest counts were different from those expected using a model with local demographic parameters suggesting a high level of immigration especially in the Ebro Delta where the difference between these two measures was larger (Fig. 6). A high level of immigration would also explain why the model failed to predict the observed fluctuations over time. This discrepancy was also found by Doxa et al. (2013) in a colony of Slender Billed gull in Southern France. Interestingly it seems that immigration at Doñana colony, at the western boundary of the species distribution, is less important because the observed and predicted dynamics had a similar trend. Our results and those from Doxa et al. (2013) suggest that even if some exchanges of individuals among the French and Spanish populations occurred, immigration from other populations outside the study area could be an important driver of Western Mediterranean population dynamics and maintenance.

Large population fluctuations are associated with increased extinction probability [2], [34]. In fact, at the local scale the confidence intervals for the estimated stochastic population growth rate were large encompassing values for a decreasing as well as increasing trend for all the studied local populations. However, the populations are connected by dispersal processes and probably these include other larger populations near the core of the species range. Consequently, at the metapopulation level the prospects for future persistence of the Slender-billed gull at the Western Mediterranean are more optimistic. This study emphasizes the necessity of studying seabird populations from a metapopulation or spatially-structured population perspective in order to understand their dynamics and to develop adequate conservation policies at large geographical scales [12], [18], [72].

## Supporting Information

Supporting Information S1 Additional tables and figures.
(DOCX)

Supporting Information S2 Details of stochastic population growth calculation.
(TXT)
