# Supplementary material for: Living on the Edge: Demography of the Slender-Billed Gull in the Western Mediterranean
Source: PLoS One. 2014 Mar 24;9(3):e92674. doi: 10.1371/journal.pone.0092674 (PMC3963922; doi:10.1371/journal.pone.0092674)
Supplement: Supporting Information S1 — Additional tables and figures. (DOCX) [file pone.0092674.s001.docx]

**SUPPORTING INFORMATION S1**

**Table S1.** Spatial distribution of Slender-billed gull resightings during breeding period.

|  | **Colony of origin** | | | |
| --- | --- | --- | --- | --- |
|  | Ebro Delta | | Doñana | |
| **Resighting location** | N | % | N | % |
| Ebro Delta | 299 | 60.8 | 4 | 0.3 |
| France | 38 | 7.7 | 2 | 0.1 |
| Levante | 45 | 9.1 | 14 | 1.0 |
| Doñana | 11 | 2.2 | 1369 | 95.9 |
| Ebro Delta & France | 32 | 6.5 | 0 | 0.0 |
| Ebro Delta & Levante | 57 | 11.6 | 1 | 0.1 |
| Ebro Delta & Doñana | 0 | 0.0 | 5 | 0.4 |
| France & Levante | 3 | 0.6 | 0 | 0.0 |
| France & Doñana | 2 | 0.4 | 4 | 0.3 |
| Levante & Doñana | 0 | 0.0 | 28 | 2.0 |
| Ebro Delta & France & Levante | 4 | 0.8 | 1 | 0.1 |
| Ebro Delta & Levante & Doñana | 1 | 0.2 | 0 | 0.0 |
| Ebro Delta total | 392 | 79.7 | 11 | 0.8 |
| France total | 80 | 16.3 | 7 | 0.5 |
| Levante total | 110 | 22.4 | 44 | 3.1 |
| Doñana total | 14 | 2.8 | 1406 | 98.5 |

Number of Slender billed gulls marked as chicks at Ebro Delta and Doñana and resighted in one or more breeding areas (Fig. 1) during breeding periods (April to July) from 1995 to 2008 (% respect the total number of birds resighted: 492 from Ebro Delta and 1428 from Doñana).

**Table S2.** Estimated annual number and % (relative to breeding pairs) of immigrant (from Ebro Delta and Doñana) Slender-billed gull at Ebro Delta and Doñana during breeding period (April to July) from 1996 to 2008.

|  | Breeding in Ebro Delta  (immigrants from Doñana) | | Breeding in Doñana  (immigrants from Ebro Delta) | |
| --- | --- | --- | --- | --- |
| **Year** | N estimated | % | N estimated | % |
| 1996 | - | - | - | - |
| 1997 | - | - | - | - |
| 1998 | - | - | - | - |
| 1999 | - | - | - | - |
| 2000 | - | - | 65 | 12.99 |
| 2001 | 8 | 0. 77 | 3 | 0. 35 |
| 2002 | - | - | 6 | 0. 87 |
| 2003 | 8 | 0. 65 | 13 | 1. 32 |
| 2004 | - | - | 2 | 0. 21 |
| 2005 | 30 | 2. 84 | 10 | 2. 01 |
| 2006 | 28 | 3.35 | 15 | 1.90 |
| 2007 | - | - | - | - |
| 2008 | 8 | 1.71 | - | - |

The annual observed number of immigrants individuals was corrected by the % of marked individuals in the natal population and recapture probabilities at breeding population following Balkiz et al. (2007). A symbol – is used when the number and proportions are not estimable (i.e., no individuals resighted this year).

**Table S3.** Annual (λ) and stochastic ($\lambda s)$ breeding population growth rates of Slender-billed gulls at Ebro Delta (EB), Doñana (DO), Levante (LE) and France (FR) regions, and in the whole Western Mediterranean region considered (WM). The R-script used to compute WM $\lambda s$ is provided in Appendix 2.

| Year | EB | DO | LE | FR | WM |
| --- | --- | --- | --- | --- | --- |
| λ 1991-1992 | 1.00 | 0.51 | - | 1.04 | 0.95 |
| λ 1992-1993 | 1.50 | 4.78 | - | 2.61 | 2.07 |
| λ 1993-1994 | 0.98 | 0.57 | - | 1.12 | 1.01 |
| λ 1994-1995 | 1.06 | 1.41 | 1.50 | 1.35 | 1.24 |
| λ 1995-1996 | 0.93 | 1.34 | 0.70 | - | - |
| λ 1996-1997 | 0.70 | 0.63 | 1.75 | - | - |
| λ 1997-1998 | 0.78 | 1.25 | 2.14 | 1.00 | 1.03 |
| λ 1998-1999 | 1.59 | 1.30 | 1.13 | 1.44 | 1.42 |
| λ 1999-2000 | 1.06 | 1.20 | 1.73 | 1.05 | 1.14 |
| λ 2000-2001 | 0.92 | 1.67 | 1.06 | 1.03 | 1.09 |
| λ 2001-2002 | 1.32 | 0.76 | 1.14 | 0.65 | 0.90 |
| λ 2002-2003 | 0.80 | 1.53 | 0.65 | 1.04 | 0.96 |
| λ 2003-2004 | 1.07 | 1.15 | 1.08 | 0.50 | 0.91 |
| λ 2004-2005 | 0.79 | 0.44 | 2.22 | 1.34 | 1.00 |
| λ 2005-2006 | 0.62 | 1.61 | 1.23 | 0.95 | 1.07 |
| λ 2006-2007 | 0.93 | 1.43 | 0.54 | 1.11 | 0.92 |
| λ 2007-2008 | 0.03 | 0.38 | 1.26 | 1.91 | 0.94 |
| λ 2008-2009 | 20.43 | 1.85 | 1.35 | 0.89 | 1.26 |
| λ 2009-2010 | 1.02 | 1.56 | 0.94 | 1.00 | 1.10 |
| λ 2010-2011 | 1.00 | 0.80 | 1.30 | 0.73 | 0.94 |
| λ 2011-2012 | 0.58 | 1.41 | 0.84 | 1.27 | 1.08 |
| Range λ 1991-2012 | 0.03-20.43 | 0.38-4.38 | 0.54-2.22 | 0.65-2.61 | 0.90-2.07 |
| $\lambda s$ | 0.95 | 1.07 | 1.19 | 1.09 | 1.08 |
| CI $\lambda s$ | 0.59-1.47 | 0.87-1.43 | 0.98-1.40 | 0.91-1.24 | 0.97-1.16 |

**Figure S1**. Estimates of age dependent resighting probabilities of Slender-billed gulls at Ebro Delta (A) and Doñana (B) breeding areas during the study period (Models 13EB and 13DO, Tables 1-2).

A)

B)
